# "The Hospital" Medical Book Supplement—No.XXVIII

**Published:** 1910-04-16

**Authors:** 


### "THE HOSPITAL" MEDICAL BOOK SUPPLEMENT—No. XXVIII.

### CONTENTS.

| Medicine— | PAGE |
|---|---|
| Public Health ... ... ... ... ... ... ... ... ... | 85 |
| Consumption, its Prevention and Home Treatment ... ... | 85 |
| The Diet Factor in Disease ... ... ... ... ... ... | 85 |
| Hoblyn's Dictionary of Terms used in Medicine and the Collateral Sciences ... ... ... ... ... ... ... ... | 85 |
| Essentials of Medical Electricity and Radiography ... ... | 85 |
| Cerebral Congestion and Tight Neck-clothing ... ... ... | 86 |
| The Medical Annual, 1910 ... ... ... ... ... ... | 86 |

| Miscellaneous— | PAGE |
|---|---|
| Nervousness ... ... ... ... ... ... ... ... | 86 |
| The Care of Children ... ... ... ... ... ... ... | 86 |
| The Hospital Service Book ... ... ... ... ... ... | 86 |
| Spiritualism and Insanity ... ... ... ... ... ... | 87 |
| A Short Handbook of Cosmetics ... ... ... ... ... | 87 |
| American Meat and its Influence upon the Public Health ... | 87 |
| Colour Blindness and Colour Perception ... ... ... ... | 87 |
| Open-Air at Home ... ... ... ... ... ... ... ... | 87 |
| The Case against Christian Science ... ... ... ... ... | 88 |
| Quack Remedies ... ... ... ... ... ... ... ... | 88 |
| The Expectation of Life of the Consumptive after Sanatorium Treatment ... ... ... ... ... ... ... ... ... | 88 |

### MEDICINE.

PUBLIC HEALTH. ("Catechist Series.") In Five Parts. By W. ROBERTSON, M.D., D.P.A. (Edinburgh: E. S. Livingstone. Price 1s. net.)

To the student who is about to take his D.P.H. diploma, these books, written on the question and answer plan, may prove useful. But they are "cram" books, and we cannot honestly recommend them to anyone desirous of thoroughly obtaining a knowledge of his subject. For to the non-studious individual there is always a desire to desert the text-book and to seek information in these homœopathic doses that are prescribed in this Catechist Series. And to our mind that should be discouraged.

CONSUMPTION, ITS PREVENTION AND HOME TREATMENT. By H. HYSLOP THOMSON, M.D. (London: Henry Frowde and Hodder and Stoughton. 2s. net.)

THE author states in his preface that this book has been written in the hope that it will prove a useful help to the consumptive and his friends in carrying out to the best possible advantage the home treatment of this disease. In all ways it is an admirable little work, and he who is unfortunate enough to be afflicted with tubercular trouble can do no better than read, mark, learn, and inwardly digest its contents, for a thorough appreciation of the rules and regulations appertaining to the "open-air" cure will go a long way to assist the patient back to health. We would direct the attention of readers of this little volume especially to one chapter—that on "Precautions against Infection." It is well written, and explains with simplicity, yet clearly, how best to prevent the spread of phthisis, and we can only say, if the suggestions offered here were more widely acted upon, consumption would become a less prevalent malady. It is a worthy little contribution to the literature on the much vexed question of the treatment of tuberculosis.

THE DIET FACTOR IN DISEASE. By GEORGE BLACK, M.B.Edin. (London: John Bale, Sons and Danielsson, Ltd. 1909. Pp. 91. Price 2s.)

THIS pamphlet, a reprint of an article which appeared in the *British Homœopathic Review*, is not as its title might suggest a study of diet in relation to disease in general. The author confines his remarks to diet in cases of appendicitis. Being a convinced vegetarian, he is in entire disagreement with ordinary methods of treatment and diet, but his most severe strictures are reserved for the surgeon, since he strongly disapproves of surgical interference, even when signs of abscess formation are evident. His faith in the adaptability of the human organism is such that he believes it is possible to cure these cases by rest, fomentations, and appropriate homœopathic medication. He quotes freely from English, American, and French writers in support of his view, and brings his work to a close by printing notes taken on ten cases which came under his own observation, and which he treated successfully by purely homœopathic and vegetarian methods.

HOBLYN'S DICTIONARY OF TERMS USED IN MEDICINE AND THE COLLATERAL SCIENCES. 14th Edition, revised and edited by J. A. P. PRICE, M.D. (London: Geo. Bell and Sons, 1909. Pp. 868. Price 10s. 6d.)

THE task of bringing up to date a standard work of reference such as Hoblyn's Dictionary must be no light one. Both in medicine and surgery, and even more so in such "collateral sciences" as bacteriology, electro-therapeutics, and so on, the number of new terms to be defined and annotated must be enormous in a few years. The task has been boldly tackled by the present editor, whose labours are everywhere apparent in the inclusion of the latest and most up-to-date researches and advances. Taking a few words at random, we found satisfactory paragraphs on Bier's treatment, Calmette's reaction, and some other recent advances. Wassermann's, and even Pirquet's and Moro's reactions, however, have escaped notice; this is a pity, because the first certainly, and probably the other two, are more likely to establish themselves permanently than is Calmette's reaction. Pellagra is said to be Italian leprosy: lupus erythematosus is classed with lupus vulgaris as a tuberculide, and the synonym of ulerythema, which some dermatologists use for it, is not recognised. These are small points: more important are that the scholarly style of the original remains unaltered or even improved, and that the publishers have done well their share of the finished product. We congratulate the editor on the result of his labours, which we are sure have not been light.

ESSENTIALS OF MEDICAL ELECTRICITY AND RADIOGRAPHY. By EDWARD REGINALD MORTON, M.D. (London: Henry Kimpton. Second Edition. 1910. Pp. 349. Price 6s. net.)

IN this second edition of Dr. Morton's manual is included a new part, almost equal in length to the original sections on medical electricity, dealing with the essentials of radiography. In these new sections the discussion of *x*-ray technique rather than the interpretation of *x*-ray pictures or the question of *x*-ray therapeutics chiefly engages attention; in thus limiting himself the author is wise, for had he been more ambitious he must have made his book either cumbrous or incomplete. Naturally the qualities and perversities of the *x*-ray tube come in for the largest share of description, and for novices the mastery of this subject is so all-important that no efforts to afford a clue to it are superfluous. In the first part, which now represents the whole of the original edition, the subject of medical electricity is dealt with in all its branches. Preliminary chapters on electricity, its forms and methods of production are very clearly written, without undue technicality, yet with all that in practice it is necessary to know concerning the properties of electricity. When subsequently the author comes to deal with the applications of electric currents and discharges in medical practice, he shows a commendable reserve in many matters. Dr. Morton is not one of those who can shut their eyes to the failures of electric methods, and he does not hesitate to indicate plainly for what complaints electric treatment has proved disappointing. If there were any criticism to make of these therapeutic chapters, one would feel inclined to regret that they are not more full, especially with regard to details of such recent developments as ionic medication, etc. Probably the author wants to be quite sure of his facts before he commits himself, and such reserve is, of course, far preferable to an unreasoning and unreasonable enthusiasm for the last new fad, whatever it may be, which specialists sometimes display.

Cerebral Congestion and Tight Neck-clothing. By Walter G. Walford, M.D., Durham. (London : H. K. Lewis, 1910. Pp. 26. 8vo. Price 1s. 6d. net.)

The author has come to the conclusion that many disorders are the outcome of the use of neck-clothing sufficiently tight to interfere with free circulation of the blood in the head and neck, and to press unduly upon the nerves of those regions. He has himself benefited to a marked degree by wearing his collars looser, and to this fact attributes his recent freedom from headache, rheumatism, high blood-pressure, and gouty manifestations, to all of which he was formerly subject. He also quotes cases in which similar good results have followed upon the use of looser neck-wear. He has the enthusiasm of the discoverer for his subject, and while being unable to agree with all his contentions and conclusions, we are ready to admit that tight collars cannot but be unhealthy, and that his method is worth trying in cases where no other obvious cause can be found to account for symptoms.

The Medical Annual, 1910. (Bristol : J. Wright and Sons. Price 8s. 6d. net.)

When an annual has reached the secure position of being indispensable, there must always be for its editor and publisher the temptation to let well alone and to drift—to rely, in fact, on the same policy that has built up past popularity. Particularly well might this be the case with the well-known compendium of the past year's progress now under review; for the task of supervising its production, even on the old-established lines, must certainly be no light one. But such a course does not satisfy the ambitions of those responsible for the *Medical Annual*; to play the safe game or to rest on their oars does not appeal to them, and the result is seen in the ever-increasing excellence of the publication.

In Part I. a large number of new synthetic and other drugs are dealt with; the claims of their makers and of those who have experimented with them are analysed. The large number of these products is evidence of the gullibility of the profession in rushing to adopt any and every new specific whose makers are unscrupulous enough to advance exaggerated or fraudulent claims of its therapeutic virtues. In this present issue alone the number of these euphoniously named patent drugs is such that no practitioner could possibly remember a quarter of them; and since experience proves that but a very tiny percentage will have any real value, the best that can be hoped is that the others will speedily follow their innumerable predecessors into the limbo of deserved oblivion. Then come articles on vaccines, by Dr. A. B. Harris; on hormones, by Dr. Emil Novak, of Baltimore; and on injections of sea water, by Dr. Robert Simon, of Paris. The latter treatment is enthusiastically eulogised by its inventor, and is spoken of as almost specific for diseases of the digestive system and for intoxications of all sorts; we confess to a large measure of scepticism, and should have preferred to see the *Medical Annual* wait a more unbiassed verdict before extending such ample hospitality to the author. This first part concludes with a review of progress in radiology and electro-therapeutics by Dr. E. R. Morton.

Part II. deals with the past year's research under the alphabetical classification of the diseases concerned, illustrated as usual with coloured plates and numerous figures in the text. The only notable omission is in respect of anæsthetics. Dr. Probyn Williams' name no longer appears on the list of contributors, and no one else seems to have been commissioned to undertake this section. The miscellaneous sections which form Part III. are more extensive and useful than ever.

### MISCELLANEOUS.

Nervousness. By Alfred T. Schofield, M.D. (London : William Rider and Son. 1s. net.)

We have carefully read this small book through, and we cannot say honestly that we are impressed by it. That nervousness is undoubtedly a disease—and to those who suffer from it a very real disease—we readily admit, and we equally disparage the medical man who insists that there is nothing the matter with the patient so afflicted; but we are at a loss to understand what would happen if the "leaders of the Emmanuel Movement"—whoever they are—were to step in and try to effect a cure. This is not a book we can recommend to practitioners.

The Care of Children. By Bernard Myers, M.D., C.M. With a preface by George F. Still, M.D., F.R.C.P. (London : Henry Kimpton. Price 1s. 6d. paper, 2s. 6d. cloth.)

Seldom has it been our good fortune to read a book for the lay reader so cleverly written and so helpful as the present one. Every mother who would rear her child to the best advantage can only do so by possessing the necessary knowledge and information. And it is just that which is to be found in the present volume. Hygiene, dietics, and sick nursing, all in their turn are dealt with, and no woman if she has a copy by her of this little work can plead ignorance of how to bring up her baby in the best possible manner. We congratulate the author on his effort.

The Hospital Service Book. By the Rev. C. P. Baxter, M.A. Revised and Enlarged. (London : Henry Frowde. 1910. Price 1s. net.)

It gives us great pleasure to call attention to this re-issue of a simple service book for which we predicted popularity on its first appearance, and to note that it has already reached its nineteenth thousand. In its present form it remains substantially what it was in the previous editions, with a few additions which further experience has dictated to the author. That it has found favour with varying schools of thought within the Church is evident from the authorship of the testimonials and letters of approval which it has evoked : and this is not to be wondered at, since it contains nothing calculated to offend any of the somewhat widely separated wings of the established religion of this country. The only criticism we have to offer is in respect of the advertisement which is allowed to appear facing the last page of the text; but as this is in respect of the hospital at which the author is chaplain, he has at least an excuse; still, we think the inclusion of this secular appeal is a mistake in a book of this kind.

Spiritualism and Insanity. By Dr. C. Williams. (The Ambrose Co., Ltd., 55 and 57 Wigmore Street, W. Price 1s. 6d. net, cloth.)

The aim of this little book is to prove that "it is the plain duty of medical men to warn people if they value their mental health to have nothing whatever to do with spiritualism." As the author himself was a spiritualist at one time, and is still a medical man, he ought to know. Like most reactionaries from any movement, his violent denunciations at the present point to his enthusiasm years ago. The book is presumably written for popular reading, and as such may serve its author's purpose, but it is based on such *a priori* grounds, and withal takes spiritualism so seriously, that we fear it will hardly appeal to the profession. The author in fact puts such a simple faith in the truth of demoniacal possession that he really pays to spiritualism what has been called the homage of revolt. We imagine the best way of combating such neurotic people as spiritualists is not to talk to them of demons so much as to expose the chicanery with which all spiritualism is bound up.

A Short Handbook of Cosmetics. Dr. Max Joseph. (London: Rebman, Ltd. Price 2s. 6d. net.)

Comparatively few medical men have any knowledge of cosmetic treatment of the skin. Yet it is distinctly a branch of medicine in which there is much scope, and the practitioner who has a knowledge of the hygiene of the skin and hair, and the treatment of their minor affections, can claim an advantage over many of his colleagues. Up to the present it has generally been left to the barber or chiropodist to treat those suffering from some slight diseases of the skin or scalp, and for the simple reason that generally the "family doctor" has no idea how to compound a hair lotion, or to give a salve for such a trivial ailment—yet most annoying to a lady—as "freckles." The result of all this has been the flooding of the markets with quack, and only too often harmful, remedies, the which would have never appeared had the teaching of cosmetics found a place in medical text-books. This book will prove useful to anyone who studies it, for it abounds in helpful ideas for treating the everyday diseases of the skin and hair. Its value is enhanced by numerous prescriptions, each of which have been proved to be efficacious in the cause assigned to it. It is a book that should be found in every practitioner's library.

American Meat and its Influence upon the Public Health. By Albert Leffingwell, M.D. (London: George Bell and Sons, 1910. Pp. 203. Price 3s. 6d. net.)

It was in 1906 that revolting disclosures concerning the stockyards of Chicago aroused indignation in this country, and the purpose of Dr. Leffingwell's book is to point out the faults there still are in connection with American beef and pork. He feels that it is absolutely essential that further reforms should be inaugurated, and his idea of one way at least in which this may be brought about seems to be by invoking the assistance of the English people. He points out that England is so extensive a purchaser of American meat and pork that the behaviour of the Meat Trust in America is influenced to no small extent by the English market; if, therefore, the English people are persuaded that there are serious faults in connection with the proper inspection of American meat from the food point of view, pressure will at once be brought to bear upon the other side and the necessary reforms will be made. This appears to be the object of the book. We think it likely that it will be read even more by the public, especially that part of the public concerned in the supply of food, than by the medical profession.

Colour Blindness and Colour Perception. By F. W. Edridge-Green, M.D., F.R.C.S. (London: Kegan Paul, Trench, Trübner, and Co. 2nd edition. 1909. Pp. 322. Price 5s.)

The widespread interest aroused recently over the case of an officer of the Mercantile Marine, who was eventually allowed by the Courts a master's certificate after being rejected several times by medical examiners as colour blind, makes the second edition of Dr. Edridge-Green's book especially timely. Those who have followed the trend of medical and physiological opinion on the very intricate and difficult subject of colour blindness will be aware that the views held by this author are not unanimously admitted by all those who have devoted time to its investigation, and that a certain amount of controversy has taken place over different sections of the various conflicting theories. The matter is, indeed, so perplexing that there is room for honest divergences of opinion, and we should be sorry to say that Dr. Edridge-Green's theories, though more widely accepted than when he first published them, are established as incontrovertible. It is, therefore, unfortunate and unbecoming that the author has permitted himself, in the preface to this new edition, to indulge in some very bitter remarks about those who do not see eye to eye with him. Accusations of prejudice and of lack of scientific spirit and open mind in opponents are apt to recoil upon the heads of those who make them. We say this not in any hostile spirit, but because a good case is here in danger of being spoilt by too polemical a method of presentation. For all who are interested in colour blindness (and since one male in seventeen is colour blind in greater or less degree, there must be few whose relatives are all normal sighted), the exposition of the subject as here presented is bound to possess a great fascination. Nor is an expert knowledge of optics or of physiology necessary for the comprehension of Dr. Edridge-Green's hypotheses. The practical sides of the question as it affects seamen, railway employees, artists, and many others, are exhaustively considered. If there is any aspect which might with advantage be amplified, it is that of inheritance. A careful study of colour blindness from the Galtonian and Mendelian standpoints would demand a separate volume to itself; but it may be hoped that when the next edition is called for the author will be able to offer at least an outline of this side of his subject. To the non-professional mind, perhaps the most striking fact quoted is the unusual prevalence of colour blindness among musicians. Can it be that their artistic instincts or perceptions, unstimulated and unappeased owing to imperfect apprehension of beauty seen, find their outlet in an increased appreciation of beauty heard? We offer the suggestion for Dr. Edridge-Green's consideration.

Open-Air at Home. By Stanley H. Bates. With introduction by Sir James Crichton-Browne, M.D. (Bristol: John Wright and Sons. Price 2s. 6d. net.)

Of recent years no treatment has come into vogue so much as the open-air treatment of consumption, and the value of so treating what has hitherto been thought a most "deadly" disease can be judged by the excellent results obtained. To those suffering from phthisis this little book will prove of real value. It is written by the lay mind, since it seeks to instruct not so much the physician as the patient. The art of building and fitting up an open-air shelter in all ways compatible with comfort and hygienic principles is fully and comprehensively treated, and the context illustrated with some excellent diagrams, and also actual photos of shelters tenanted by patients. It is a small book whose usefulness is beyond question.

The Case against Christian Science. By Stephen Paget, F.R.C.S. (London: Cassell and Co. 1909. Price 6d. net.)

Quack Remedies. By David Walsh, M.D.(Edin.) (London: Baillière, Tindall, and Cox. 1909. Price 1s. 6d. net.)

We group these two unpretending, but valuable, pamphlets together, because in their different ways each is doing a public service by calling attention to a public nuisance. Mr. Paget's papers were read to the Church Congress at Swansea and the Congregational Union at Sheffield. They are distinguished, it is scarcely necessary to say, by the literary finish and graceful style which we have come to expect of him. His demonstration of the utter absence of logic, sense, and common decency in the fundamental tenets of Mrs. Eddy's system, when once they are stripped of the magniloquent verbiage with which she and her prominent disciples clothe them, is a fine piece of penetrating philosophical analysis; but it wants very careful and leisured reading, and is—we suggest it with deference—more suited to print than to a public platform, for which reason the present publication is very welcome. It is otherwise with the brief but eloquent passages in which are summed up the results of Christian Science teachings as investigated by experts; in them the fraud, the cruelty, the unnecessary misery and suffering caused by Mrs. Eddy are denounced in a few burning sentences, which stamp Mr. Paget an orator as well as a philosopher. We suppose in this era of political corruption and title-selling it is useless to suggest to the King's advisers that one who has devoted years to the service and protection of the public, and especially the ignorant public, against the harpies who exploit it, deserves at least as much recognition as the moneyed nonentities who contribute to party funds. Nevertheless, the view is widely held, and we shall be bold enough to uphold it against all comers; though we do not feel equally sure that we should advise so worthy a son of so eminent a father to consent to be herded with the motley crowd of company-promoting aliens, party agents, and wirepullers generally that is contained in honour lists nowadays.

Dr. Walsh's reprint of a series of articles which first obtained publicity in the columns of the *Medical Press and Circular* is equally welcome and timely. Based largely upon the invaluable publication, *Secret Remedies*, of the British Medical Association, and upon similar exposures in other countries (often Governmental reports), it is well calculated to drive home the moral contained in the information which is afforded by the bulkier volumes. The author's suggestion of an *index expurgatorius* of newspapers which have refused to review the British Medical Association's publication, or have refused the advertisement thereof, and, at the same time, draw revenues running into five and six figures annually from the advertisement of quack medicines and patently dishonest cure-alls, is unfortunately more praiseworthy than practical. Nothing reveals more fully the state of corruption of the public press in this country than the disgraceful episodes in connection with *Secret Remedies*; and it would not greatly surprise us to find them repeated in the case of Dr. Walsh's book. We quote from the *British Medical Journal* of October 16, 1909, five newspapers by which the advertisement in question was refused: the *Daily Express, Daily Chronicle, Star, Graphic, News of the World*. The *Daily Mail* at first declined, but afterwards repented and accepted. The *Daily Telegraph, Punch, Spectator, Manchester Guardian*, and a few others published the advertisement readily, to their honour; and we are very pleased to note that the *Spectator* has recently (December 18) reviewed the book at considerable length. For drawing attention once more to this grave scandal the thanks of every honest man are due to Dr. Walsh. The only criticism we should like to offer on his book is in respect of the somewhat inferior paper and type with which it is produced.

The Expectation of Life of the Consumptive after Sanatorium Treatment. By Noel D. Bardswell. (London: Messrs. Henry Frowde and Hodder and Stoughton. 1910. Pp. 130. Price 3s. 6d. net.)

The book is arranged in three main parts, the first of which is a summary of the results of treatment, arranged mainly in the form of tables. The third part from p. 37 to p. 130 consists of the abstracts of the clinical notes and after-histories of 241 individual cases. Between these two comes what may be described as the kernel of the work—namely, a commentary on the cases (p. 30 to p. 36). To statisticians and to specialists both the tables of results and the summaries of case-histories will be of interest, but to the general practitioner the book consists mainly of the six pages of commentary. The value of the suggestions made in this commentary, however, is great, and the author's remarks are most practical and to the point. He lays particular stress upon the fact that the immediate benefit of sanatorium treatment is so great in many cases that patients and their friends are apt to be deceived by appearances, thinking themselves so completely cured in a short time that they fail to take proper precautions during the succeeding months and years, and therefore speedily relapse. Complete cure is not likely to take place within two years of what may be termed arrest. It is clearly impossible for a patient to stay all that time in a sanatorium, so that it is essential to regard sanatorium treatment as only the first step in the cure. Discussing the question of whether the patient may remain too long in a sanatorium, Dr. Bardswell lays stress on the fact that many people are apt to become introspective and anxious, and to lose self-reliance out of all proportion as to what is justified by their state of health; such patients should cease sanatorium treatment at once. He also discusses the question of occupation, and points out how very difficult it is for wage-earners to change their mode of livelihood, so that the treatment subsequent to living at a sanatorium has to be a compromise between an ideal open-air existence and the necessary application to business. His experience of sea voyages has not been altogether satisfactory. He points out how great a feature is temperament or character in determining the ultimate success or failure. To the consumptive who possesses earnestness of purpose, common-sense, courage, and patience cure is much more probable than to one who lacks these characteristics. The cultivation of cheerfulness is therefore an essential part of the treatment. Notwithstanding this, the general conclusion come to is that sanatoria by no means reduce the mortality from phthisis in any degree that is remarkable; there is a reduction, but unless the open-air treatment or a modification of it can be continued after the sanatorium is left, patients are apt to do badly even when they have been temporarily relieved.

